# Neutrophil-to-albumin ratio predicts long-term prognosis in coronary heart disease: a prospective cohort study of 2,990 patients

**DOI:** 10.3389/fnut.2025.1674969

**Published:** 2025-12-17

**Authors:** Hao Wu, Kaiyue Feng, Yiming Hua, Zehao Lin, Ning Ding, Yifei Xie, Yu Xu, Yue Wu, Danyu Cheng

**Affiliations:** 1 Department of Cardiovascular Medicine, First Affiliated Hospital, Xi’an Jiaotong University, Xi’an, Shaanxi, China; 2 Key Laboratory of Molecular Cardiology, Xi’an, Shaanxi, China; 3 Key Laboratory of Environment and Genes Related to Diseases, Ministry of Education, Xi’an, Shaanxi, China

**Keywords:** coronary heart disease, major adverse cardiovascular events, albumin, neutrophil percentage, prognosis

## Abstract

**Background:**

Cardiovascular diseases (CVDs), particularly coronary heart disease (CHD), impose a global health burden with unpredictable major adverse cardiovascular events (MACE) despite optimal treatment. Systemic inflammation and malnutrition are key pathogenic drivers, yet integrated biomarkers capturing this duality are lacking. The Neutrophil-to-Albumin Ratio (NPAR) emerges as a cost-effective indicator reflecting both pathways, but its prognostic value for post-discharge MACE in hospitalized CHD patients remains unestablished. Our study aimed at investigating the prognostic value of NPAR for MACE in CHD patients.

**Methods:**

This prospective cohort study enrolled CHD patients (2013–2020) from the First Affiliated Hospital of Xi’an Jiaotong University. NPAR was calculated from admission blood samples. Participants were stratified into NPAR tertiles (T1: 5.21–16.00; T2: 16.01–18.50; T3: 18.51–42.38). Multivariable Cox models (adjusting for demographics, comorbidities, and laboratory parameters) assessed NPAR-MACE associations. Restricted cubic splines (RCS) and subgroup analyses explored nonlinearity and effect modifiers.

**Results:**

A total of 2,990 patients with CHD were eligible for analysis. With a median follow-up of 70 months, Kaplan–Meier survival curves demonstrated that participants in the highest NPAR tertile (T3) exhibited significantly lower cumulative survival rates free from MACE compared to those in the lowest tertile (T1). The highest NPAR tertile (T3) exhibited a 72% increased MACE risk (adjusted hazard ratio (HR) = 1.72, 95% confidence interval (CI): 1.36–2.20) versus T1. A J-shaped relationship emerged, with risk escalating above NPAR = 17 (P nonlinear = 0.01). Sex-specific heterogeneity was observed: males in T3 had markedly elevated risk (HR = 2.03, 95% CI: 1.50–2.75; P interaction = 0.03).

**Conclusion:**

Elevated admission NPAR independently predicts long-term MACE in CHD patients, particularly among males. This supports NPAR’s utility for post-discharge risk stratification.

## Graphical Abstract

**Figure:**
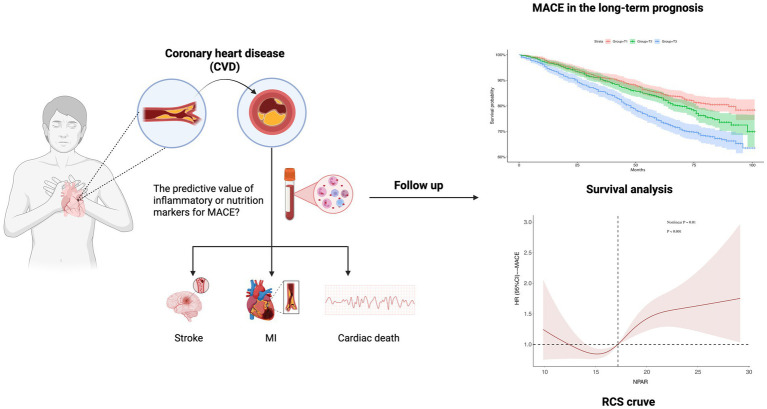
Admission NPAR independently predicts long-term MACE in CHD patients. Graphical abstract demonstrates the strong clinical predictive performance of the neutrophil-to-albumin ratio (NPAR) for long-term major adverse cardiovascular events (MACE) in patients with coronary heart disease (CHD) after discharge. Created in BioRender. Xie, Y. (2025) https://BioRender.com/4ismqah.

## 1 Introduction

Cardiovascular diseases (CVD) represent the leading cause of death globally, accounting for over one-third of all deaths annually (1). According to estimates from the Global Burden of Disease (GBD) Study, CVD were responsible for approximately 17.8 million deaths worldwide in 2017. This figure indicates a 21% increase in CVD mortality over the preceding decade leading up to 2017. Within this burden, ischemic heart disease (IHD) and stroke contributed to nearly 50 and 35% of CVD deaths, respectively (2). Coronary heart disease (CHD), the primary underlying cause of IHD, stands as one of the major cardiovascular conditions threatening global human health. By 2019, heart disease had become the foremost contributor to disability-adjusted life years in individuals aged 50 years and older (3).

The progression of CHD is dynamic and unpredictable, potentially precipitating unexpected major adverse cardiovascular events (MACE), such as myocardial infarction (MI), revascularization, heart failure (HF), stroke, and cardiovascular death. A critical concern is that the risk of MACE remains high even among patients who have undergone revascularization and are receiving guideline-directed optimal secondary prevention (4, 5). Consequently, there is a compelling need for enhanced risk stratification models that incorporate sensitive biomarkers and clinical indicators. Such models are essential for identifying high-risk patients, thereby enabling precision secondary prevention strategies for CHD.

While recent studies have explored risk stratification for Major Adverse Cardiovascular Events (MACE) in various clinical contexts—such as systematic reviews of conventional risk factors post-PCI (6), inflammatory markers in the elderly (7), perioperative outcomes in CAD patients (8), and traditional predictors in post-MI cohorts (9)—these approaches often rely on established risk factors requiring complex assessment, focus on specific subpopulations, or are derived from retrospective analyses. In contrast, our study introduces a novel perspective by prospectively validating the Neutrophil-to-Albumin Ratio (NPAR)—a simple, cost-effective, and integrative inflammatory-nutritional biomarker—in a large, broadly representative CHD cohort with long-term follow-up. This approach provides a readily accessible tool for predicting long-term post-discharge MACE, addressing a critical gap between conventional risk evaluation and the need for efficient, scalable risk stratification in routine practice.

Nutritional status is closely associated with CVD risk. Pathophysiological alterations triggered by decreased serum albumin concentration promote the development of CVD. Research indicates that lower serum albumin levels are primarily linked to malnutrition and inflammatory responses, and are significantly associated with adverse clinical outcomes in patients with CHD (10). Systemic inflammation is increasingly recognized as a pivotal factor in the initiation and progression of chronic diseases (11). Low-grade, chronic systemic inflammation significantly impacts long-term disease prognosis and outcomes (12), underscoring the crucial value of inflammation-related biomarkers in long-term prognostic assessment and management. Inflammation is now considered a key driver in the pathogenesis of CVD. While classic inflammatory markers such as white blood cell count and C-reactive protein (CRP) serve as established predictors of disease prognosis, they exhibit certain limitations in sensitivity and specificity (13). Robust evidence confirms the intimate link between inflammation and atherosclerosis, highlighting its critical role in coronary plaque progression and adverse events following stent implantation (14, 15).

Traditional biomarkers often struggle to reflect the complex interplay between nutrition and inflammation (16). More importantly, there is a current lack of integrated biomarkers capable of simultaneously and comprehensively assessing both inflammatory and nutritional status, which is crucial for a more holistic understanding of CVD risk. The neutrophil percentage-to-albumin ratio (NPAR) has recently garnered attention as a novel composite indicator integrating inflammation and nutritional status. Neutrophils play a vital role in the immune system, primarily through phagocytosis of pathogens and clearance of damaged cells (17). An elevated neutrophil percentage typically signifies an inflammatory state, particularly evident in chronic diseases or acute stress conditions like infection and tissue injury. Albumin, the most abundant plasma protein, serves not only as an indicator of nutritional status but also possesses diverse physiological functions, including anti-inflammatory, antioxidant, and anticoagulant properties (18). Studies demonstrate a significant association between low albumin levels and both chronic inflammation and malnutrition (19). By assessing inflammation via neutrophils and nutritional status via albumin, NPAR provides a more comprehensive evaluation of a patient’s condition than single biomarkers.

The clinical utility of NPAR has been validated in several fields. Research indicates its prognostic value in conditions such as cancer, HF, and chronic obstructive pulmonary disease (20–22). A growing body of evidence supports the clinical significance of the NPAR within specific disease contexts. However, evidence regarding the association between the NPAR and cardiovascular outcomes, particularly its ability to predict long-term prognosis after discharge in hospitalized CHD patients, remains limited. Therefore, this study utilizes data from CHD patients hospitalized in our center between 2013 and 2020, followed over the long term, to evaluate the predictive value of the NPAR for the probability of MACE occurring after discharge.

## 2 Materials and methods

### 2.1 Study population and design

#### 2.1.1 Study population

This study enrolled 4,399 patients diagnosed with CHD who were admitted to the Department of Cardiology, First Affiliated Hospital of Xi’an Jiaotong University between January 2013 and July 2020. After applying exclusion criteria, data from 2,990 patients (2,182 males and 808 females) aged 28 to 89 years were included in the final analysis. Patients underwent a median follow-up period of 75 months. The diagnosis of CHD was established based on coronary angiography results reviewed by at least two experienced interventional cardiologists, confirming the presence of ≥50% stenosis in at least one major coronary artery. All patients received treatment according to standard clinical guidelines. The study was conducted in accordance with the Declaration of Helsinki and received approval from the Ethics Committee of the First Affiliated Hospital of Xi’an Jiaotong University. Written informed consent was obtained from all participants prior to inclusion.

#### 2.1.2 Exclusion criteria

Patients were categorized into three groups based on their calculated NPAR values. Exclusion criteria were: (1) Lack of laboratory data required for NPAR calculation; (2) Concurrent diagnosis of acute or chronic infectious disease upon admission; (3) History of repeated hospitalizations; and (4) Concurrent diagnosis of cancer.

#### 2.1.3 Data collection

Detailed demographic information, clinical data, medication history, and laboratory test results (including blood biochemistry) were extracted from inpatient medical records. Demographic variables included age, sex, and race/ethnicity. History of comorbidities (including cancer, diabetes, hypertension, dyslipidemia), smoking and alcohol consumption status, and relevant medication use were collected via patient self-reporting. Body weight and height were measured to calculate body mass index (BMI = weight [kg] / height^2^ [m^2^]). Post-discharge follow-up was conducted via telephone interviews to ascertain survival status, readmission history, and the occurrence of MACE.

### 2.2 Measurement of NPAR

Complete blood count parameters, including cell counts and size analysis, were determined using the Beckman Coulter method. Albumin levels were measured using the LX20 analyzer employing the bichromatic digital endpoint method. For the calculation of the NPAR, the neutrophil percentage (representing the proportion of neutrophils within the total white blood cell count) was first determined. NPAR was calculated using the following formula applied to the same blood sample: NPAR = [Neutrophil Percentage (%)] / [Albumin (g/dL)] (22, 23). NPAR was analyzed both as a continuous variable and categorized into tertiles.

### 2.3 Statistical analysis

Continuous variables are summarized as mean ± standard deviation, and categorical variables as frequency (percentage). Group comparisons across NPAR tertiles were performed using one-way ANOVA for continuous variables and Pearson’s chi-square test for categorical variables. Cox proportional hazards regression models were utilized to assess the association between three biomarkers and Major Adverse Cardiovascular Events (MACE), calculating hazard ratios (HRs) with 95% confidence intervals (CIs). Indicators was analyzed both as a continuous variable and in tertile categories (T1–T3), with the lowest tertile (T1) serving as the reference group across all models.

Three sequential models were constructed to adjust for potential confounders. Model 1: Unadjusted. Model 2: Adjusted for demographic factors: age, sex, and BMI. Model 3: Fully adjusted for demographics, comorbidities (diabetes mellitus, history of ACEI/ARB use), clinical vital signs (systolic and diastolic blood pressure), and laboratory parameters (blood glucose, LDL-C, TG, TC, CRE, ALT, AST, Ca^2+^, K^+^).

Several supplementary analyses were conducted to characterize the predictive properties of the biomarkers based on the fully adjusted Model 3. Restricted cubic spline (RCS) regression analyses were performed to examine the potential non-linear associations between each continuous biomarker (ALB, NLR, and NPAR) and the risk of MACE. The incremental predictive value of adding each biomarker to the baseline clinical model (Model 3) was evaluated and compared. This assessment was based on changes in the C-statistic, the Net Reclassification Index (NRI), and the Integrated Discrimination Improvement (IDI). To assess the robustness and consistency of the primary association, subgroup analyses for NPAR (as a continuous variable) were performed across prespecified patient strata, including sex, age (≤65 vs. >65 years), BMI (<25 vs. ≥25 kg/m^2^), comorbidities (hypertension, diabetes mellitus), and smoking status, using the fully adjusted Model 3.

## 3 Results

### 3.1 Baseline data

**Figure 1:**
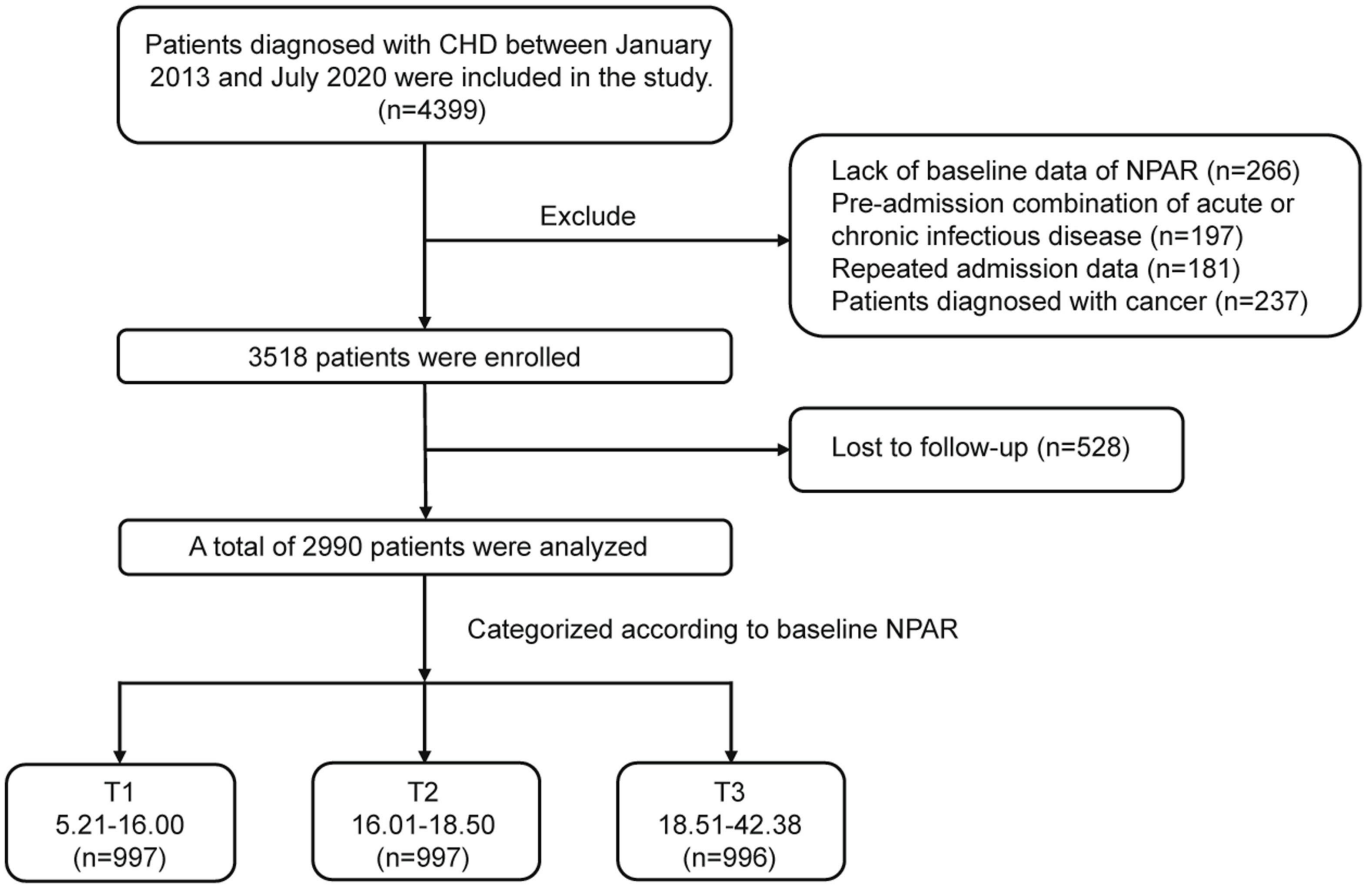
Overall flowchart of this study. From an initial screening of 4,399 patients with CHD, 2990 were included in the final analysis after implementing exclusion criteria and addressing loss to follow-up. CHD, coronary heart disease; NPAR, neutrophil-to-albumin ratio.

**Figure 2:**
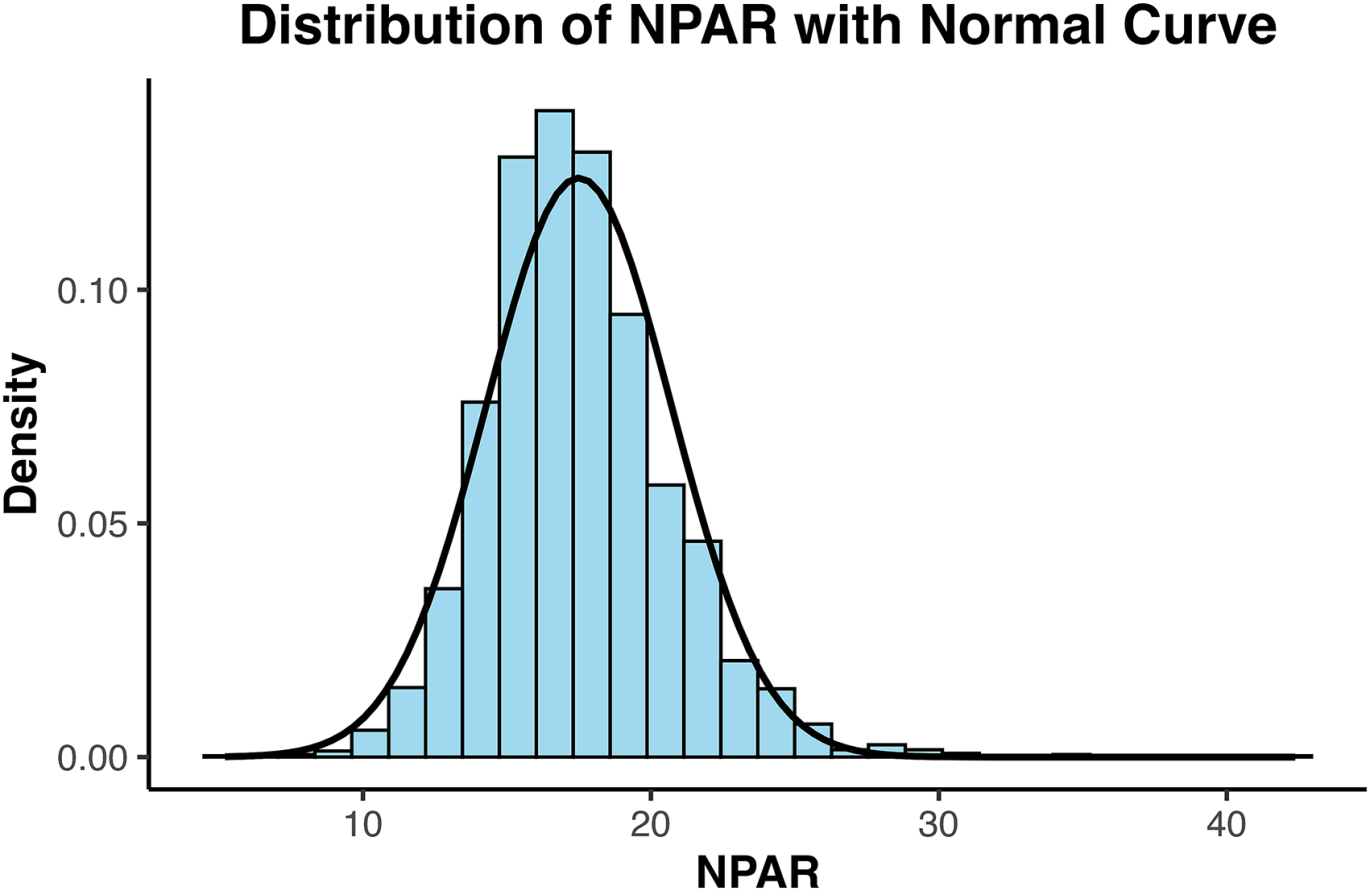
Distribution of baseline NPAR levels in the cohort. NPAR, neutrophil-to-albumin ratio.

A total of 2,990 patients were ultimately included in the study and stratified into three groups based on NPAR levels: T1 (NPAR: 5.21–16.00), T2 (NPAR: 16.01–18.50), and T3 (NPAR: 18.51–42.38). The flowchart of this study is shown in Figure 1. The baseline NPAR levels in the overall study population demonstrates an approximately normal distribution (Figure 2). Regarding baseline demographic characteristics, significant differences were observed among the three groups for sex, age, and BMI. Specifically, the T3 group had the highest proportion of male patients and the oldest mean age compared to both the T1 and T2 groups. Analysis of underlying comorbidities revealed no significant difference in the prevalence of hypertension across the groups. However, significant differences were observed in the prevalence of diabetes mellitus and smoking status. The T3 group exhibited the highest prevalence of diabetes mellitus and the highest proportion of smokers. Significant differences were also found in the history of ACEI/ARB medication use between the groups. In contrast, no significant differences were observed in the proportions of patients taking aspirin, Plavix, *β*-blocker, or statins. Significant differences were present among the groups for both blood pressure (systolic and diastolic) and blood glucose levels. Among the laboratory parameters, significant differences were detected for LDL-C, TG, TC, CRE, ALT, AST, Ca2^+^, and K^+^. No significant differences were found for high-density lipoprotein cholesterol (HDL-C) or blood urea nitrogen (BUN). Baseline characteristics according to the NPAR tertiles is shown in Table 1.

**Table 1:** Baseline characteristics according to the NPAR tertiles.

Characteristic	T1	T2	T3	*p*-value
	*N* = 997	*N* = 997	*N* = 996	
Gender (%)				<0.001
Male	684 (69%)	718 (72%)	780 (78%)	
Female	313 (31%)	279 (28%)	216 (22%)	
Age, year	60.25 ± 9.48	61.64 ± 10.11	62.93 ± 10.87	<0.001
BMI, kg/m^2^	24.66 ± 3.00	24.46 ± 3.06	24.13 ± 3.16	0.001
Hypertension (%)	582 (58%)	578 (58%)	560 (56%)	0.6
Diabetes Mellitus (%)	543 (54%)	584 (59%)	637 (64%)	<0.001
Smoker (%)	348 (35%)	352 (35%)	384 (39%)	0.2
Aspirin (%)	911 (91%)	925 (93%)	905 (91%)	0.3
Plavix (%)	627 (63%)	659 (66%)	670 (67%)	0.10
β-blocker (%)	784 (79%)	795 (80%)	794 (80%)	0.8
ACEI/ARB (%)	797 (80%)	822 (82%)	739 (74%)	<0.001
Statin (%)	907 (91%)	915 (92%)	890 (89%)	0.2
SBP, mmHg	132.98 ± 18.64	132.40 ± 20.09	128.77 ± 21.02	<0.001
DBP, mmHg	78.02 ± 11.35	77.41 ± 11.42	76.52 ± 12.40	0.013
Glucose, mmol/L	6.90 ± 2.96	7.19 ± 3.21	7.69 ± 4.10	<0.001
LDL-cholesterol, mmol/L	2.33 ± 0.83	2.24 ± 0.84	2.23 ± 0.78	0.022
HDL-cholesterol, mmol/L	0.99 ± 0.24	0.97 ± 0.24	0.98 ± 0.25	0.084
Triglycerides, mmol/L	1.87 ± 1.47	1.70 ± 1.08	1.43 ± 0.85	<0.001
Cholesterol, mmol/L	3.97 ± 0.98	3.84 ± 0.98	3.82 ± 0.93	<0.001
BUN, mmol/L	5.53 ± 1.51	5.48 ± 1.75	5.69 ± 2.23	0.3
Creatinine, μmol/L	65.42 ± 28.31	66.73 ± 28.98	69.95 ± 26.81	<0.001
ALT, U/L	31.51 ± 27.38	30.11 ± 27.00	40.96 ± 61.23	<0.001
AST, U/L	29.80 ± 27.65	37.25 ± 47.65	69.95 ± 111.17	<0.001
Calcium, mmol/L	2.29 ± 0.12	2.26 ± 0.13	2.21 ± 0.15	<0.001
Potassium, mmol/L	4.04 ± 0.39	4.01 ± 0.42	3.96 ± 0.44	<0.001
MACE events	178 (18%)	221 (22%)	305 (31%)	<0.001

BMI, body mass index; SBP, systolic blood pressure; DBP, diastolic blood pressure; LDL-cholesterol, low-density lipoprotein cholesterol; HDL-cholesterol, high-density lipoprotein cholesterol; BUN, blood urea nitrogen; ALT, alanine aminotransferase; AST, aspartate aminotransferase; MACE, major adverse cardiovascular events.

### 3.2 Clinical outcome

**Figure 3:**
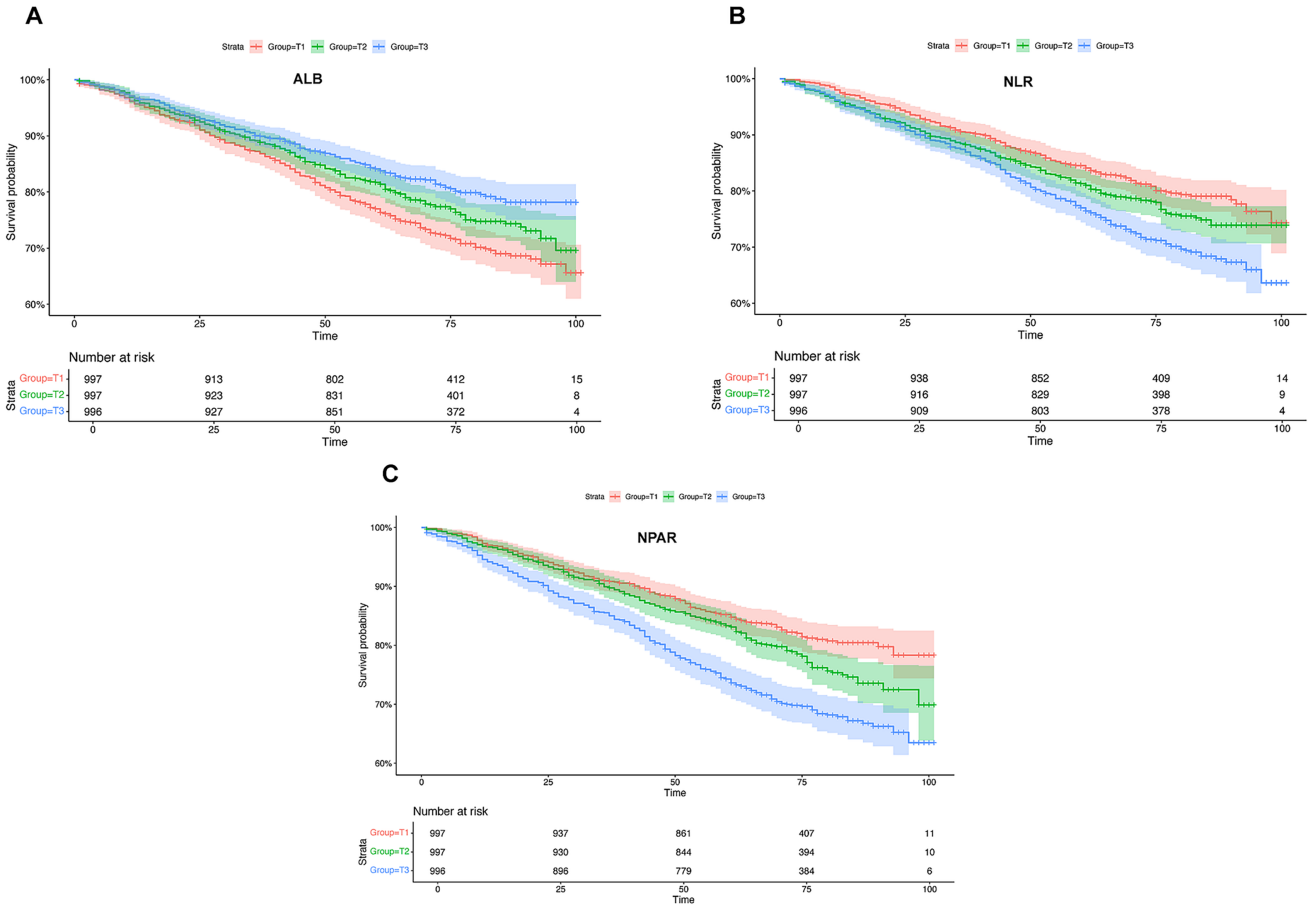
Kaplan–Meier survival curves for MACE. Complex survey-weighted Kaplan–Meier survival curves for **(A)** ALB; **(B)** NLR and **(C)** NPAR by tertiles. This figure highlights prognostic utility of three indicators across MACE risk. MACE, major adverse cardiovascular events; ALB, albumin; NLR, neutrophil-to-lymphocyte ratio; NPAR, neutrophil-to-albumin ratio.

After a median follow-up period of 75 months, the cumulative incidence of MACE was 18% in the T1 group, 22% in the T2 group, and 31% in the T3 group (*p* < 0.001). This demonstrates a significantly increased risk of MACE in patients within the highest NPAR tertile (T3) (Table 1). Consistent with this finding, Kaplan–Meier survival analysis revealed that participants in the highest NPAR tertile (T3) exhibited significantly lower cumulative survival rates free from MACE compared to those in the lowest tertile (T1) (Figure 3). We also performed Kaplan–Meier survival analysis for two other traditional biomarkers related to inflammation and nutrition, neutrophil-to-lymphocyte ratio (NLR) and albumin (ALB). The results showed that the group with the highest NLR demonstrated lower cumulative MACE-free survival rates, while the group with the highest ALB levels exhibited higher cumulative MACE-free survival rates.

### 3.3 Association of predictive factors with MACE

Three Cox proportional hazards regression models were employed to investigate the independent relationship between ALB, NLR and NPAR levels with MACE events. Based on the Cox regression analyses (Table 2), all the factors demonstrated significant associations with MACE risk, though with distinct patterns across adjustment models.

**Table 2:** COX regression of the association between three biomarkers and MACE.

Categories	Model 1	*P* value	Model 2	*P* value	Model 3	*P* value
	HR (95%CI)		HR (95%CI)		HR (95%CI)	
ALB						
ALB as continuous	0.94 (0.93, 0.96)	<0.001	0.97 (0.94, 0.99)	0.003	0.96 (0.93, 0.99)	0.01
Tertile 1	Ref		Ref		Ref	
Tertile 2	0.81 (0.68, 0.96)	0.01	0.92 (0.76, 1.11)	0.4	0.89 (0.71, 1.11)	0.28
Tertile 3	0.65 (0.54, 0.78)	<0.001	0.78 (0.64, 0.96)	0.02	0.80 (0.63, 1.03)	0.08
NLR						
NLR as continuous	1.05 (1.03, 1.07)	<0.001	1.05 (1.02, 1.07)	<0.001	1.05 (1.02, 1.08)	0.001
Tertile 1	Ref		Ref		Ref	
Tertile 2	1.21 (1.00, 1.47)	0.05	1.14 (0.93, 1.41)	0.22	1.04 (0.83, 1.31)	0.73
Tertile 3	1.57 (1.30, 1.88)	<0.001	1.52 (1.24, 1.85)	<0.001	1.37 (1.09, 1.73)	0.007
NPAR						
NPAR as continuous	1.08 (1.06, 1.10)	<0.001	1.06 (1.03, 1.08)	<0.001	1.06 (1.03, 1.10)	<0.001
Tertile 1	Ref		Ref		Ref	
Tertile 2	1.27 (1.04, 1.54)	0.02	1.22 (0.99, 1.51)	0.07	1.19 (0.94, 1.51)	0.15
Tertile 3	1.83 (1.52, 2.20)	<0.001	1.71 (1.39, 2.10)	<0.001	1.72 (1.36, 2.20)	<0.001

Model 1 was unadjusted. Model 2 was adjusted for sex, age and BMI. Model 3 was adjusted for sex, age, BMI, diabetes mellitus, history of ACEI/ARB medication use, SBP, DBP, blood glucose concentration, LDL-C, TG, TC, creatinine, ALT, AST, Ca^2+^ and K^+^. ALB, albumin; NLR, neutrophil-to-lymphocyte ratio; NPAR, neutrophil-to-albumin ratio; MACE, major adverse cardiovascular events; HR, hazard ratio; CI, confidence interval.

For albumin levels, a clear inverse relationship with MACE risk was observed. Although the highest albumin tertile showed progressively attenuated protective effects with increasing model adjustments, it consistently demonstrated the lowest MACE risk across all models, with the fully adjusted Model 3 showing a trend toward protection (HR 0.80, 95% CI 0.63–1.03, *p* = 0.08). Conversely, NLR exhibited a positive association with MACE risk. In the fully adjusted Model 3, participants in the highest NLR tertile demonstrated a 37% increased risk of MACE (HR 1.37, 95% CI 1.09–1.73, *p* = 0.007) compared to the lowest tertile. What’s more, NPAR levels also demonstrated a significant association with increased MACE risk. Compared to the reference group (T1, lowest NPAR tertile): the T2 tertile group had an HR of 1.19 (95% CI: 0.94, 1.51), the T3 tertile group (highest NPAR) had an HR of 1.72 (95% CI: 1.36, 2.20). The overall association across NPAR tertiles was statistically significant (*p* < 0.001). When NPAR is treated as a continuous variable, the HR for MACE risk was 1.06 (95% CI: 1.03, 1.10), which was also statistically significant (*p* < 0.001). In summary, the Cox regression analyses demonstrate that all three biomarkers—albumin, NLR, and NPAR—serve as independent predictors of long-term MACE risk. Particularly noteworthy are the substantial risk elevations observed in the highest tertiles of both inflammatory markers. Patients in the top NLR tertile experienced a 37% increased risk of MACE (HR 1.37, 95% CI 1.09–1.73), while those in the highest NPAR tertile faced an even more pronounced 72% risk elevation (HR 1.72, 95% CI 1.36–2.20) in the fully adjusted model.

**Figure 4:**
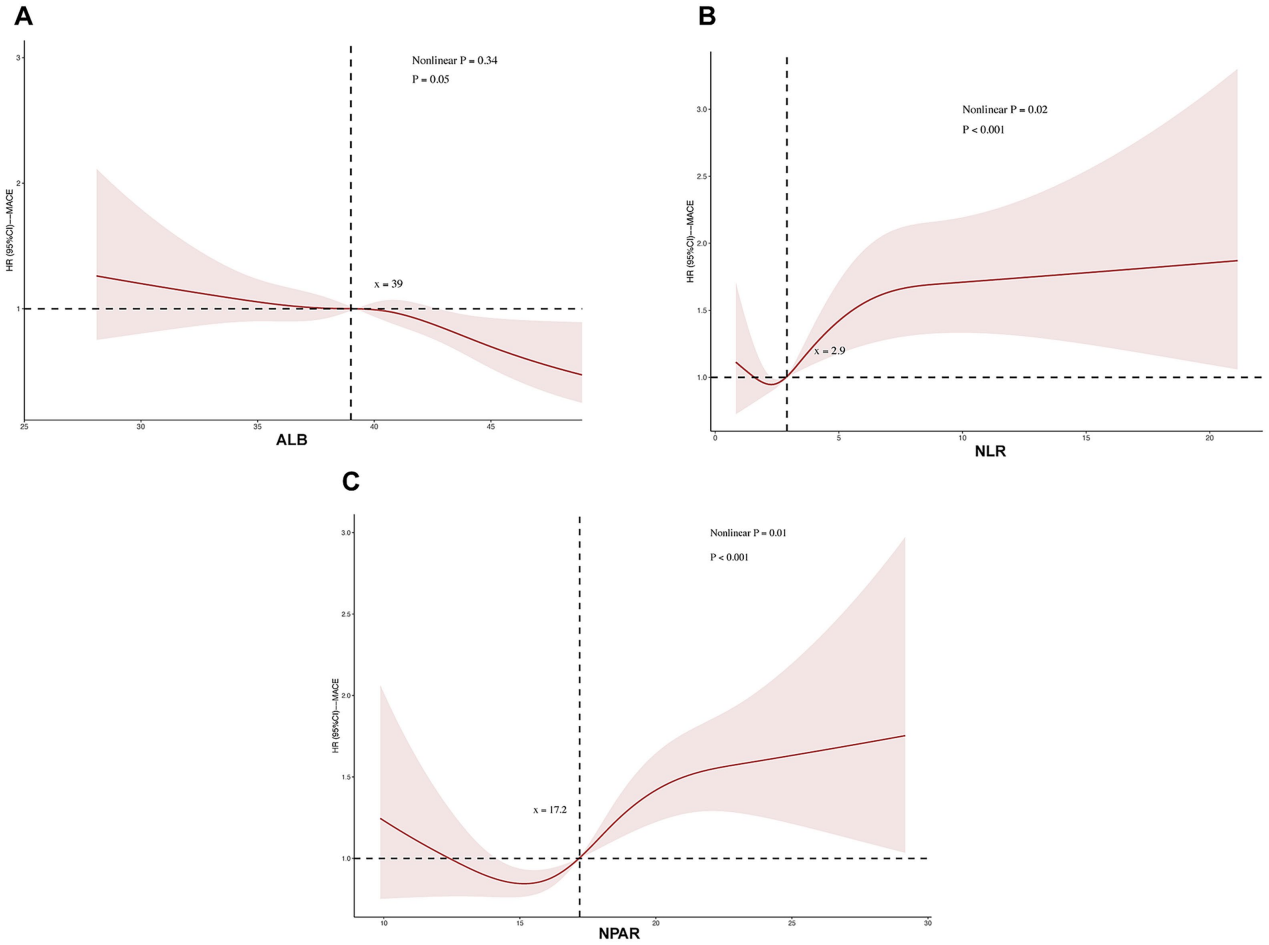
Restricted cubic spline (RCS) analyses were performed to test nonlinear associations. Restricted cubic spline analysis of the nonlinear association between **(A)** ALB, **(B)** NLR, **(C)** NPAR and MACE in CHD patients. For **(A)** albumin, the relationship with MACE risk followed a linear pattern (p for nonlinear = 0.34). In contrast, both **(B)** NLR (p for nonlinear = 0.02) and **(C)** NPAR (p for nonlinear = 0.01) demonstrated significant nonlinear relationships with MACE risk. Notes: The results were adjusted for sex, age, BMI, diabetes mellitus, history of ACEI/ARB medication use, SBP, DBP, blood glucose concentration, LDL-C, TG, TC, creatinine, ALT, AST, Ca^2+^ and K^+^. ALB, albumin; NLR, neutrophil-to-lymphocyte ratio; NPAR, neutrophil-to-albumin ratio; MACE, major adverse cardiovascular events.

To comprehensively evaluate the shape of association between inflammatory-nutritional biomarkers and MACE risk, we performed restricted cubic spline (RCS) analyses based on the fully adjusted Model 3 (Figure 4). The RCS curves revealed distinct association patterns for the three biomarkers. For albumin, the relationship with MACE risk followed a linear pattern (p for nonlinearity = 0.34; p for overall association < 0.001), with risk progressively decreasing as albumin levels increased across the entire range of values. In contrast, both NLR and NPAR demonstrated significant nonlinear relationships with MACE risk. For NLR, the association was nonlinear (p for nonlinearity = 0.02; p for overall association < 0.001), with a threshold effect observed around NLR = 2.9. Similarly, NPAR exhibited a pronounced nonlinear association with MACE risk (p for nonlinearity = 0.01; p for overall association < 0.001). The RCS curve identified an inflection point at NPAR = 17.2, below which MACE risk showed minimal variation, but beyond which risk escalated sharply with increasing NPAR values. These threshold-based relationships for both NLR and NPAR provide important clinical insights, suggesting specific cutoff values that may be useful for risk stratification in clinical practice.

### 3.4 Incremental prognostic value of NPAR

To evaluate the incremental predictive value of NPAR beyond traditional risk factors, improvements in risk reclassification and model discrimination were assessed using NRI and IDI metrics. The baseline model included established clinical risk factors (Model 3) (Table 3).

**Table 3:** Reclassification statistics (95% CI) and integrated discrimination improvement to comprehensive evaluation of the predictive performance of NPAR.

Model	C-statistics (95%CI)	*P* value	Continuous NRI (95%CI)	*P* value	IDI (95%CI)	*P* value
Model3	0.5818 (0.5295–0.6343)	0.002	Ref		Ref	
Model3 + ALB	0.5879 (0.5367–0.6390)	<0.001	0.089 (−0.003–0.197)	ns	0.002 (0.000–0.005)	0.092
Model3 + NLR	0.5949 (0.5425–0.6472)	<0.001	0.170 (0.046–0.255)	<0.001	0.002 (0.000–0.007)	0.064
Model3 + NPAR	0.6016 (0.5500–0.6532)	<0.001	0.275 (0.173–0.373)	<0.001	0.003 (0.000–0.010)	0.028

Model 3 was adjusted for sex, age, BMI, diabetes mellitus, history of ACEI/ARB medication use, SBP, DBP, blood glucose concentration, LDL-C, TG, TC, creatinine, ALT, AST, Ca 2^+^ and K^+^. ALB, albumin; NLR, neutrophil-to-lymphocyte ratio; NPAR, neutrophil-to-albumin ratio; NRI, Net Reclassification Index; IDI, integrated discrimination improvement; CI, confidence interval.

In terms of model discrimination, NPAR showed the highest improvement in C-statistics (0.6016, 95% CI: 0.5500–0.6532, *p* < 0.001) compared to the baseline model (0.5818, 95% CI: 0.5295–0.6343, *p* = 0.002), albumin (0.5879, 95% CI: 0.5367–0.6390, *p* < 0.001), and NLR (0.5949, 95% CI: 0.5425–0.6472, *p* < 0.001). The addition of NPAR to the baseline model resulted in substantial improvement in risk reclassification for MACE (NRI = 0.275, 95% CI: 0.173–0.373, *p* < 0.001), significantly outperforming both ALB alone (NRI = 0.089, 95% CI: −0.003–0.197, *p* = NS) and NLR alone (NRI = 0.170, 95% CI: 0.046–0.255, *p* < 0.001). This demonstrates that the composite biomarker NPAR provides superior reclassification ability compared to its individual components. For integrated discrimination improvement, NPAR demonstrated significant enhancement (IDI = 0.003, 95% CI: 0.000–0.010, *p* = 0.028), while albumin and NLR showed non-significant improvements (albumin: IDI = 0.002, *p* = 0.092; NLR: IDI = 0.002, *p* = 0.064).

These findings indicate that NPAR, by integrating both inflammatory and nutritional pathways, provides substantial incremental predictive value for MACE risk stratification beyond traditional risk factors, with performance superior to either component alone.

### 3.5 Subgroup analyses

**Figure 5:**
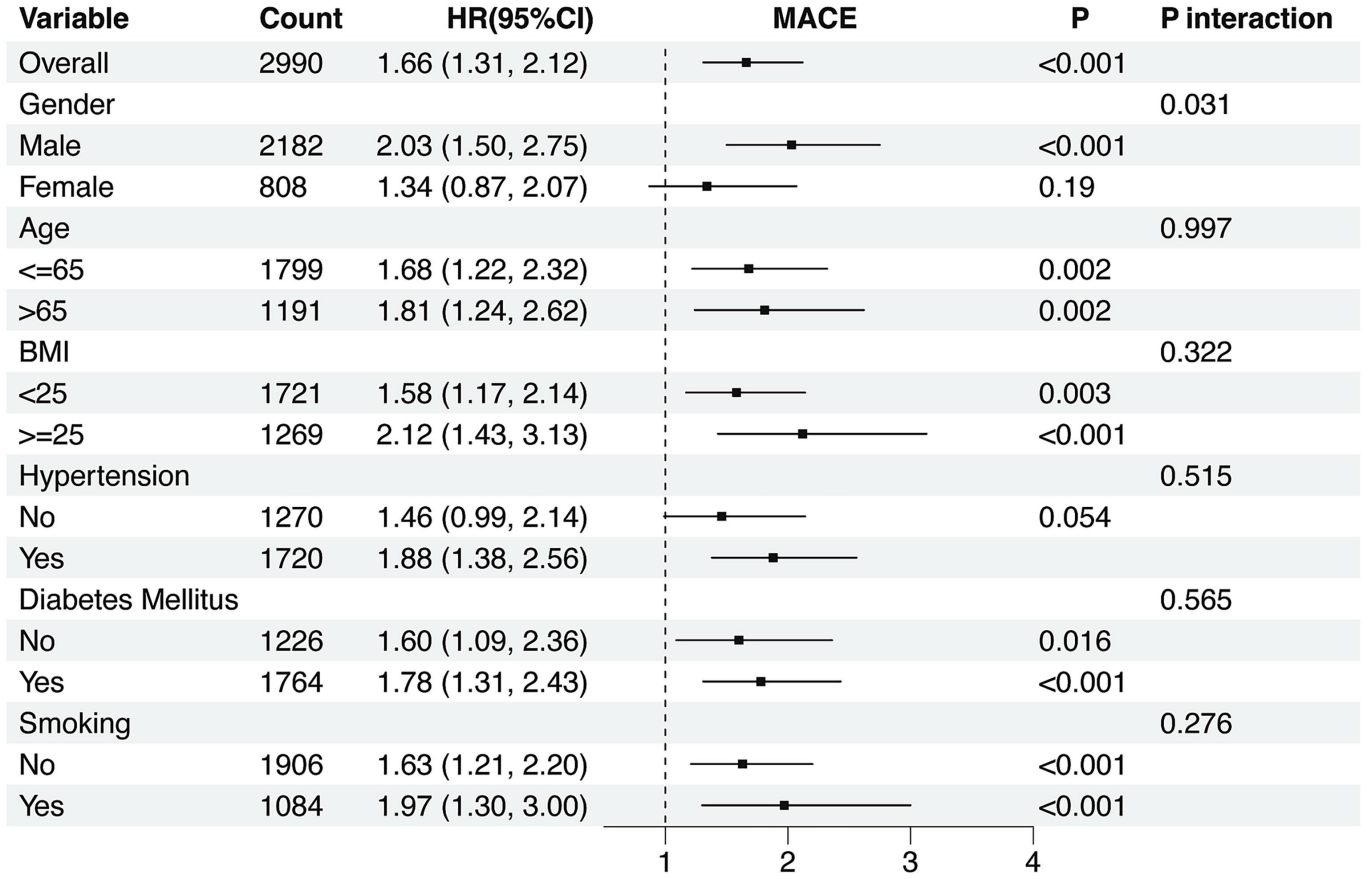
Subgroup analyses for the association of NPAR with MACE. The association between elevated NPAR and increased MACE risk remained consistent across most subgroups. Notably, a significant interaction was observed in the sex-stratified analysis (p for interaction = 0.031). Notes: Data are presented as HR (95% CI). HRs were adjusted for sex, age, BMI, diabetes mellitus, history of ACEI/ARB medication use, SBP, DBP, blood glucose concentration, LDL-C, TG, TC, creatinine, ALT, AST, Ca^2+^ and K^+^. NPAR, neutrophil-to-albumin ratio; MACE, major adverse cardiovascular events.

To further elucidate the relationship between NPAR and the risk of MACE, subgroup analyses were performed across various strata, including sex, age, BMI, hypertension, diabetes mellitus, and smoking status. The association between elevated NPAR and increased MACE risk remained consistent across most subgroups. Notably, a significant interaction was observed in the sex-stratified analysis (p for interaction = 0.031). In males, higher NPAR levels were significantly associated with an increased risk of MACE (HR = 2.03, 95% CI: 1.50–2.75), whereas in females, although the hazard ratio showed an increasing trend (HR = 1.34, 95% CI: 0.87–2.07), the association was not statistically significant. No significant interactions were found in other subgroups, suggesting that sex may serve as a potential effect modifier in the relationship between NPAR and adverse cardiovascular outcomes (Figure 5).

## 4 Discussion

### 4.1 NPAR independently predicts long-term MACE events in CHD

This study evaluated the impact of the NPAR measured at hospital admission on long-term MACE in patients with CHD (median follow-up: 75 months). The results demonstrated that patients with higher NPAR levels had a significantly increased risk of post-discharge MACE compared to those in the lowest NPAR group. Specifically, after adjusting for covariates including sex, age, BMI, diabetes mellitus, history of ACEI/ARB medication use, SBP, DBP, blood glucose concentration, LDL-C, TG, TC, CRE, ALT, AST, Ca^2+^, K^+^, the highest NLR tertile group exhibited a hazard ratio for MACE of 1.37 (95% CI 1.09–1.73), while NPAR in the highest tertile group exhibited a more pronounced 72% risk for MACE (HR = 1.72, 95% CI: 1.36–2.20). Most importantly, our findings strongly establish the superior predictive utility of NPAR, the composite index integrating both nutritional and inflammatory pathways. NPAR not only demonstrated the strongest association with MACE risk among the three biomarkers but also provided significant incremental predictive value over its individual components. This was evidenced by its substantially higher net reclassification improvement (NRI = 0.275) compared to albumin alone (NRI = 0.089) or NLR alone (NRI = 0.170), along with superior discrimination improvement as measured by IDI. The nonlinear relationship of NPAR, characterized by a distinct risk threshold at 17.2, provides a clinically actionable cutoff for risk stratification.

Subgroup analyses revealed a significant interaction effect with sex. Among male patients, the highest NPAR tertile was associated with a substantially increased MACE risk (HR = 2.03, 95% CI: 1.50–2.75). In contrast, this association was not significant among female patients (HR = 1.34, 95% CI: 0.87–2.10). What’s more, no significant interaction effects were detected in other subgroups, including age (≤65 vs. >65 years), BMI (<25 vs. ≥25 kg/m^2^), hypertension, diabetes mellitus, or smoking status.

### 4.2 NPAR in cardiovascular diseases: current evidence

CVD represent the leading global disease burden, and their incidence is closely linked to systemic inflammation. Consequently, researchers have increasingly focused on the predictive value of the NPAR for CVD onset. For instance, Peng et al. conducted a cross-sectional study using the NHANES database, revealing that higher NPAR levels were significantly associated with increased CVD risk. Compared to the lowest quartile, the highest NPAR quartile group exhibited substantially elevated risks of HF (OR = 1.66, 95% CI: 1.18–2.34) and stroke (OR = 1.74, 95% CI: 1.28–2.36) (24). Similarly, Zheng et al. analyzed NHANES data over a 20-year follow-up period and found that individuals in the highest NPAR tertile had significantly increased risks of all-cause mortality (HR = 1.45, 95% CI: 1.33–1.57) and cardiovascular mortality (HR = 1.69, 95% CI: 1.39–2.06) (25). Collectively, these findings demonstrate that NPAR is robustly associated with CVD incidence and adverse prognosis in general populations. Notably, in diabetic populations—where hyperglycemia heightens infection susceptibility—multiple studies have explored NPAR’s prognostic relevance. One recent research indicates that compared to the lowest NPAR group, the highest NPAR group had an all-cause mortality HR of 1.62 (95% CI: 1.36–1.94) and a CVD mortality HR of 1.41 (95% CI: 0.99–2.00) (26). Elevated NPAR significantly correlated with all-cause and CVD mortality in diabetic/prediabetic patients, suggesting its utility as a prognostic biomarker. The critical question remains whether NPAR can independently predict long-term outcomes in established CVD patients. Studies confirm that in hypertensive populations, higher NPAR levels markedly increased risks of all-cause mortality (HR = 1.80, 95% CI: 1.54–2.12) and cardiovascular mortality (HR = 1.54, 95% CI: 1.24–1.91) (27). Furthermore, analysis of the MIMIC-IV database involving 2,813 atrial fibrillation patients showed that elevated NPAR was independently associated with higher 30-day (OR = 2.08, 95% CI: 1.58–2.75), 90-day (OR = 2.07, 95% CI: 1.61–2.67), and 1-year mortality (OR = 1.60, 95% CI: 1.26–2.04) (28). In CHD patients, NPAR independently predicted 30-day, 60-day, and 365-day all-cause mortality in critically ill cohorts (29).

While these studies establish NPAR’s long-term predictive value for CVD events, significant knowledge gaps persist. Existing evidence primarily derives from large databases focusing on non-hospitalized populations or ICU patients. Crucially, whether NPAR predicts long-term prognosis in hospitalized CVD patients post-treatment remain unexplored. To address this, our study enrolled CHD patients admitted to the First Affiliated Hospital of Xi’an Jiaotong University (2013–2020) with a median follow-up of 75 months. We aimed to determine if NPAR could predict post-discharge adverse cardiovascular events. Our results demonstrate that elevated NPAR levels are significantly associated with increased long-term MACE incidence, underscoring its prognostic value for risk stratification in CHD patients.

### 4.3 The integrated value of NPAR: mechanisms and predictive superiority

Based on our findings and existing literature, the neutrophil-to-albumin ratio (NPAR) demonstrates considerable promise as an integrative biomarker that synergistically captures both systemic inflammatory activity and nutritional status. Its clinical value is further enhanced by its cost-effectiveness and routine accessibility. Elevated NPAR reflects neutrophilia, indicating amplified inflammatory responses, combined with hypoalbuminemia, suggesting either malnutrition or inflammation-driven catabolic processes. This dual-pathway approach enables NPAR to surpass single-dimensional biomarkers in capturing the complex pathophysiology underlying chronic cardiovascular diseases.

The biological plausibility of NPAR’s predictive capacity is rooted in well-established mechanisms linking systemic inflammation to cardiovascular pathogenesis (30). At vascular inflammation sites, activated neutrophils employ multiple synergistic strategies: releasing reactive oxygen species (ROS), secreting proteases (including cathepsin G, neutrophil elastase, and MPO) through degranulation, releasing pro-inflammatory alarmins (such as S100A8/A9), and forming neutrophil extracellular traps (NETs). These processes collectively promote atherosclerosis, plaque destabilization, and increased cardiovascular risk (31). Excessive ROS production dysregulates endothelial cell activation, degrades underlying extracellular matrix, promotes monocyte adhesion and recruitment, and facilitates LDL translocation from the lumen to the arterial intima (32). Subsequent ROS-mediated LDL oxidation and potential activation of matrix metalloproteinases may further contribute to plaque rupture (33). Myeloperoxidase indirectly impairs endothelial function by interfering with nitric oxide metabolism—a crucial element in vascular homeostasis (34). Additionally, NETs trigger interferon responses and, through NLRP3 inflammasome activation, promote macrophage secretion of IL-1β and IL-18, establishing a positive feedback loop that amplifies NET formation (35). The resulting pro-inflammatory environment, characterized by secretion of MPO, ROS, and cytotoxic histone H4, fosters conditions conducive to plaque instability and rupture while promoting vascular smooth muscle cell death. Clinically, the significance of inflammatory pathways in cardiovascular disease is well substantiated. Multiple studies have established the prognostic value of inflammatory markers—including total leukocyte count, neutrophil count, and CRP—in predicting adverse cardiovascular outcomes (36, 37). The landmark CANTOS trial further demonstrated that targeted anti-inflammatory therapy with canakinumab significantly reduced major adverse cardiovascular events (MACE) in post-myocardial infarction patients with persistent inflammation, as evidenced by elevated high-sensitivity CRP levels (>2 mg/L) (38). This provides compelling evidence that modulation of inflammatory pathways can directly improve cardiovascular outcomes.

Complementing the inflammatory axis, albumin serves as a crucial biomarker of nutritional status and possesses significant anti-inflammatory properties (39). Hypoalbuminemia is strongly associated not only with malnutrition but also with exacerbated inflammatory responses. Normal albumin levels have been shown to suppress the expression and transport of inflammatory cytokines, thereby curbing the inflammatory cascade (40, 41). Furthermore, plasma albumin binds free fatty acids (each molecule capable of binding 2–6 moles of non-esterified fatty acids) (42), participating in fatty acid and triglyceride regulation that subsequently influences cardiovascular disease progression. In clinical settings, low albumin levels consistently correlate with increased mortality risk among hospitalized patients (43).

Our results provide compelling evidence that NPAR’s composite nature translates to superior predictive performance compared to its individual components. The fully adjusted Cox regression models revealed that patients in the highest NPAR tertile faced a 72% increased risk of MACE (HR 1.72, 95% CI 1.36–2.20), substantially exceeding the risk associated with the highest NLR tertile (37% increase) and demonstrating more robust prediction than albumin alone. This substantial risk elevation suggests synergistic interaction between the inflammatory and nutritional pathways captured by NPAR. Further supporting its clinical utility, restricted cubic spline analysis identified a pronounced nonlinear relationship between NPAR and MACE risk (p for nonlinearity = 0.01) with an inflection point at NPAR = 17.2. Beyond this threshold, MACE risk escalated sharply, providing a clinically actionable cutoff for risk stratification. Most importantly, NPAR demonstrated superior incremental predictive value beyond traditional risk factors, showing the highest improvement in C-statistics (0.6016 vs. 0.5818 for baseline) and substantial improvement in risk reclassification (NRI = 0.275), significantly outperforming both ALB alone (NRI = 0.089) and NLR alone (NRI = 0.170).

Therefore, NPAR’s value extends beyond simply combining two biomarkers—it integrates neutrophilia-driven vascular injury with albumin-modulated metabolic and anti-inflammatory homeostasis into a single metric that more comprehensively reflects the complex pathophysiology underlying cardiovascular disease progression. Our findings position NPAR as a pragmatically useful tool for risk stratification, particularly valuable in populations where both inflammatory and nutritional pathways contribute to long-term cardiovascular risk.

### 4.4 Sex differences in the predictive effect of NPAR

A significant sex-specific interaction was observed in our subgroup analysis, with female patients exhibiting a markedly attenuated risk for MACE compared to males. This protective effect in females may be attributed to several interrelated biological mechanisms:

Sex Hormone-Mediated Cardio-protection: Endogenous estrogen in premenopausal women exerts multiple beneficial effects, including enhanced endothelial function via stimulation of nitric oxide production (44), favorable modulation of lipid metabolism, and suppression of pro-inflammatory pathways (45–47). Sexual Dimorphism in Immune Response: Females typically mount a more robust innate and adaptive immune response. While this increases susceptibility to autoimmune conditions, it may also promote more effective resolution of inflammation and confer differential responses to atherosclerotic stimuli compared to males (48, 49). Body Composition and Fat Distribution: The typically higher proportion of visceral adipose tissue in males is a potent source of systemic pro-inflammatory cytokines, which may amplify the inflammatory burden represented by an elevated NPAR, thereby contributing to a higher cardiovascular risk (50, 51).

In summary, the synergistic effects of cardioprotective sex hormones, distinct immune regulation, and more favorable body fat distribution likely provide women with greater physiological resilience to the “inflammatory-malnutrition” stress captured by NPAR, explaining its stronger predictive performance in men.

### 4.5 Strengths and limitations of the study

The principal innovation of this study lies in its unique design that effectively bridges a critical methodological gap in NPAR research. While previous studies have primarily relied on either large heterogeneous databases with limited follow-up or smaller single-center cohorts with restricted generalizability, our study integrates three distinctive strengths: (1) a large-scale prospective cohort with consecutive enrollment based on rigorous coronary angiography confirmation over seven years, ensuring diagnostic precision and clinical homogeneity; (2) extended follow-up (median 75 months) specifically focused on post-discharge outcomes, providing robust validation of NPAR’s long-term predictive value beyond short-term endpoints commonly reported in database studies; and (3) standardized laboratory measurements within a single-center framework, minimizing inter-institutional variability that often compromises biomarker validation. This synergistic combination of diagnostic rigor, longitudinal perspective, and methodological consistency represents a significant advancement in establishing NPAR as a reliable prognostic biomarker for long-term risk stratification in CHD patients.

However, several limitations warrant acknowledgment: as a single-center study, generalizability to other ethnicities and regions requires validation through multi-center research; NPAR was measured only once during hospitalization without post-discharge dynamic monitoring, which restricts insight into temporal variability; data integrity relied on medical records, potentially introducing documentation gaps; most critically, the observational design precludes causal inference regarding whether elevated NPAR directly increases mortality or merely reflects underlying pathologies; and despite adjusting for demographic, clinical, and lifestyle confounders, residual confounding (e.g., physical activity, protein intake, or systemic inflammatory comorbidities) cannot be fully excluded. Therefore, prospective studies remain essential to validate these findings.

While our results highlight NPAR’s clinical potential, we acknowledge that these findings require external validation across diverse populations and healthcare settings. Future studies should focus on multi-center replication to confirm the generalizability of the NPAR threshold. Furthermore, investigating the integration of NPAR with other established or novel biomarkers within multi-marker panels may yield even more accurate risk stratification models for clinical use.

## 5 Conclusion

In this large prospective cohort study of 2,990 CHD patients with a 75-month median follow-up, elevated admission NPAR independently predicted increased long-term risk of MACE. After fully multivariable adjustment, patients in the highest NPAR tertile exhibited a 72% higher risk of MACE (HR = 1.72, 95% CI: 1.36–2.20) compared to the lowest tertile. Notably, this association demonstrated significant sex-specific heterogeneity, with substantially greater risk observed in males (T3 vs. T1 HR = 2.03, 95% CI: 1.50–2.75; P-interaction = 0.03). What’s more, based on our RCS analysis which identified NPAR >17.2 as a critical risk threshold, we propose that CHD patients with admission NPAR values exceeding this cutoff should be classified as having a high risk for long-term MACE. For these high-risk individuals, we recommend implementing more intensive post-discharge surveillance, including structured follow-up protocols and enhanced patient education on risk factor management, to potentially mitigate future cardiovascular events.

These findings establish NPAR—an accessible, cost-effective biomarker integrating inflammatory and nutritional pathways—as a robust prognostic tool for post-discharge risk stratification in CHD patients. Its clinical utility is particularly pronounced in male populations, potentially guiding targeted interventions such as intensified anti-inflammatory therapy or nutritional support. While validation in multi-ethnic cohorts is warranted, this study advances personalized cardiovascular risk prediction by leveraging dual-pathophysiological insights into inflammation-nutrition interplay.

## Data Availability

The original contributions presented in the study are included in the article/supplementary material, further inquiries can be directed to the corresponding authors.
